# Melatonin and Its Metabolites Ameliorate UVR-Induced Mitochondrial Oxidative Stress in Human MNT-1 Melanoma Cells

**DOI:** 10.3390/ijms19123786

**Published:** 2018-11-28

**Authors:** Konrad Kleszczyński, Bernadetta Bilska, Agatha Stegemann, Damian Jozef Flis, Wieslaw Ziolkowski, Elżbieta Pyza, Thomas A. Luger, Russel J. Reiter, Markus Böhm, Andrzej T. Slominski

**Affiliations:** 1 Department of Dermatology, University of Münster, Von-Esmarch-Str. 58, 48149 Münster, Germany; agathastegemann@gmail.com (A.S.); thomas.luger@ukmuenster.de (T.A.L.); bohmm@uni-muenster.de (M.B.); 2 Department of Cell Biology and Imaging, Institute of Zoology and Biomedical Research, Jagiellonian University, Gronostajowa 9, 30-387 Kraków, Poland; bernadetta.bilska@doctoral.uj.edu.pl (B.B.); elzbieta.pyza@uj.edu.pl (E.P.); 3 Department of Bioenergetics and Nutrition, Gdańsk University of Physical Education and Sport, Górski Str. 1, 80-336 Gdańsk, Poland; damian.flis@awf.gda.pl (D.J.F.); wieslaw.ziolkowski@awf.gda.pl (W.Z.); 4 Department of Cellular and Structural Biology, UT Health Science Center, San Antonio, TX 78229, USA; reiter@uthscsa.edu (R.J.R.); 5 Department of Dermatology, Comprehensive Cancer Center, University of Alabama at Birmingham, Birmingham, AL 35294, USA; 6 Pathology and Laboratory Medicine Service, VA Medical Center, Birmingham, AL 35249, USA

**Keywords:** metabolites of melatonin, melanoma cells, mitochondria, catalase, oxidative phosphorylation, calcium homeostasis, ultraviolet radiation

## Abstract

Melatonin (Mel) is the major biologically active molecule secreted by the pineal gland. Mel and its metabolites, 6-hydroxymelatonin (6(OH)Mel) and 5-methoxytryptamine (5-MT), possess a variety of functions, including the scavenging of free radicals and the induction of protective or reparative mechanisms in the cell. Their amphiphilic character allows them to cross cellular membranes and reach subcellular organelles, including the mitochondria. Herein, the action of Mel, 6(OH)Mel, and 5-MT in human MNT-1 melanoma cells against ultraviolet B (UVB) radiation was investigated. The dose of 50 mJ/cm^2^ caused a significant reduction of cell viability up to 48%, while investigated compounds counteracted this deleterious effect. UVB exposure increased catalase activity and led to a simultaneous Ca^++^ influx (16%), while tested compounds prevented these disturbances. Additional analysis focused on mitochondrial respiration performed in isolated mitochondria from the liver of BALB/cJ mice where Mel, 6(OH)Mel, and 5-MT significantly enhanced the oxidative phosphorylation at the dose of 10^−6^ M with lower effects seen at 10^−9^ or 10^−4^ M. In conclusion, Mel, 6(OH)Mel and 5-MT protect MNT-1 cells, which express melatonin receptors (MT1 and MT2) against UVB-induced oxidative stress and mitochondrial dysfunction, including the uncoupling of oxidative phosphorylation.

## 1. Introduction

Melatonin (*N*-acetyl-5-methoxytryptamine) is a ubiquitous physiological mediator that is found throughout the evolutionary scale of animals and is also in plants and unicellular organisms [1,2,3,4,5]. In mammals, it is characterized as a natural neurohormone synthesized in the pineal gland, which is involved in the regulation of circadian rhythms [4]. In addition, many other tissues and cells, including bone marrow [6], lymphocytes [7], retina [8], astrocytes [9], thymus [10], skin [11,12,13], and female reproductive organs (granulosa cells, cumulus cells, and oocytes) [14], can synthesize melatonin. There is also evidence that melatonin is present in follicular fluid [15], and that it can be synthesized by oocytes [16,17]. Chemically, melatonin can function as an endogenous free radical scavenger and a broad spectrum antioxidant, and it can easily reach all cellular compartments because of its well-described amphiphilic nature [18,19,20]. Indeed, melatonin is ubiquitously localized in the cytosolic, membranous, mitochondrial, and nuclear compartments of the cell [21,22]. Interestingly, the highest melatonin concentrations are found in mitochondria [23], raising the possibility of the functional significance for this targeting with involvement in mitochondrial activities. For instance, most apoptotic signals originate in the mitochondria, and melatonin has well-known anti-apoptotic [24,25,26], anti-inflammatory [27] and prodifferentiation effects [28,29,30]. Mitochondria are also the organelles with the highest production rate of reactive oxygen/nitrogen species (ROS/RNS), which are attenuated by melatonin [31] through stimulation of the activity of anti-oxidative enzymes such as catalase (CAT), glutathione peroxidase (GPx), superoxide dismutase (SOD) [32], heme oxygenase-1 (HO-1), γ-glutamylcysteine synthetase (γ-GCS), and NADPH:quinone dehydrogenase-1 (NQO-1), which were induced via activation of nuclear erythroid 2-related factor (Nrf2) [26,33]. UVB itself affects Nrf2 and suppresses Nrf2-dependent gene expression in human skin [34]. It is recognized that mitochondria are the main target of the environmental stress factors, one of them represented by ultraviolet radiation (UVR). Exposure of the skin to UVB induces direct DNA damage [35] such as cyclobutane pyrimidine dimers (CPD) and pyrimidine photoproducts (6-4-PPs) [36,37,38,39] with the additional production of reactive oxygen species (ROS) induced by UVA [40,41]. Collectively, these changes have detrimental effects that include carcinogenesis, cell senescence, and other skin pathologies [42]. Many melatonin actions are mediated through its interaction with membrane bound type 1 and 2 (MT1 and MT2) receptors or through receptor-independent mechanisms [30,43]. However, the metabolites of melatonin can also function as antioxidants [26,33,44], and according to some authors as pro-oxidants [45]. 

Since melatonin interacts with lipid bilayers, herein on one hand, we investigated the action of melatonin and its selected metabolites under UVR-induced stress conditions. On the other hand, we tested the effect of compounds themselves on the bioenergetics of native isolated mitochondria evaluating their respiratory capacity to understand a “real face” of melatonin’s capability to stabilize mitochondrial homeostasis.

## 2. Results

### 2.1. Melatonin and Its Metabolites Mitigate UVB-Induced Cell Death 

**Figure 1:**
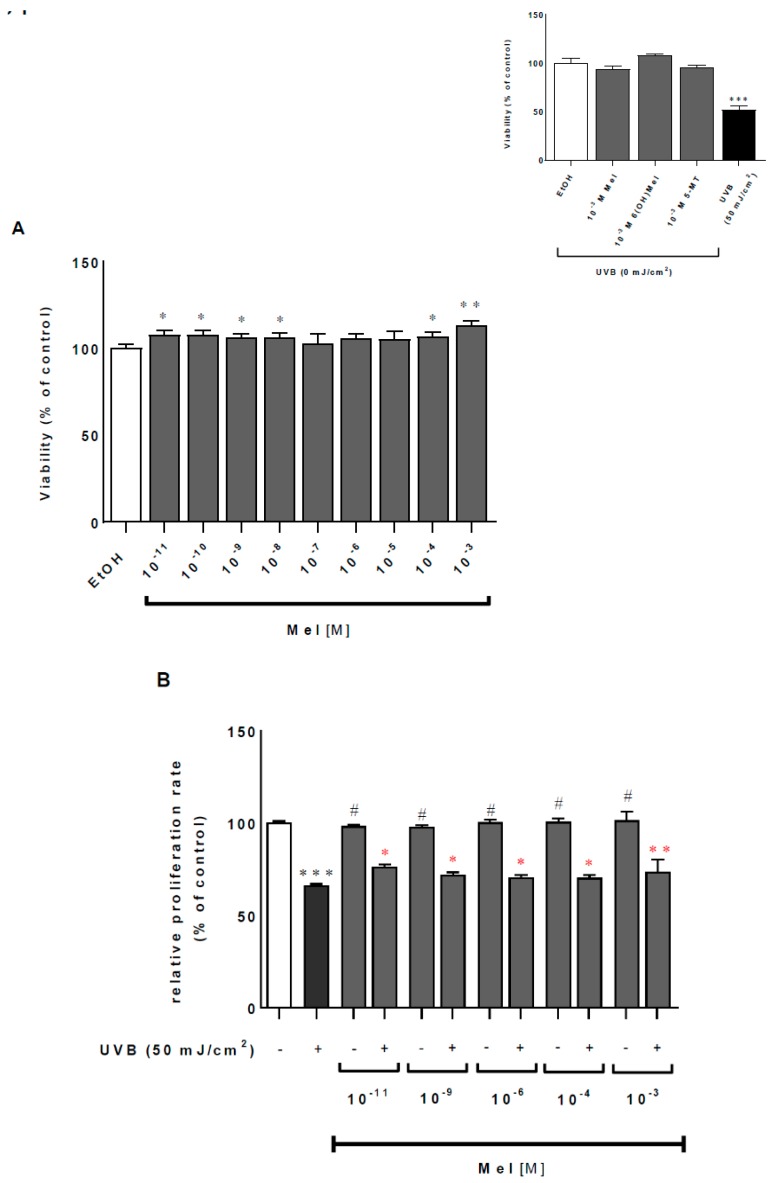

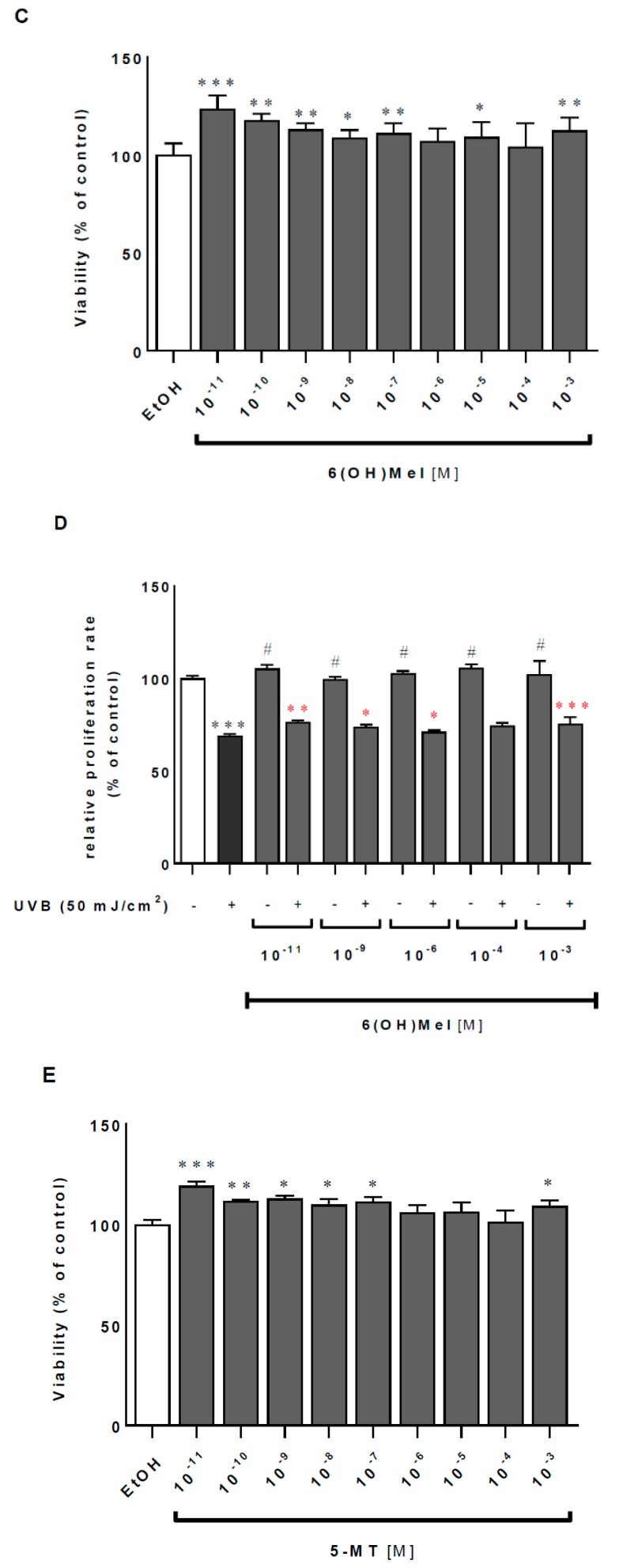

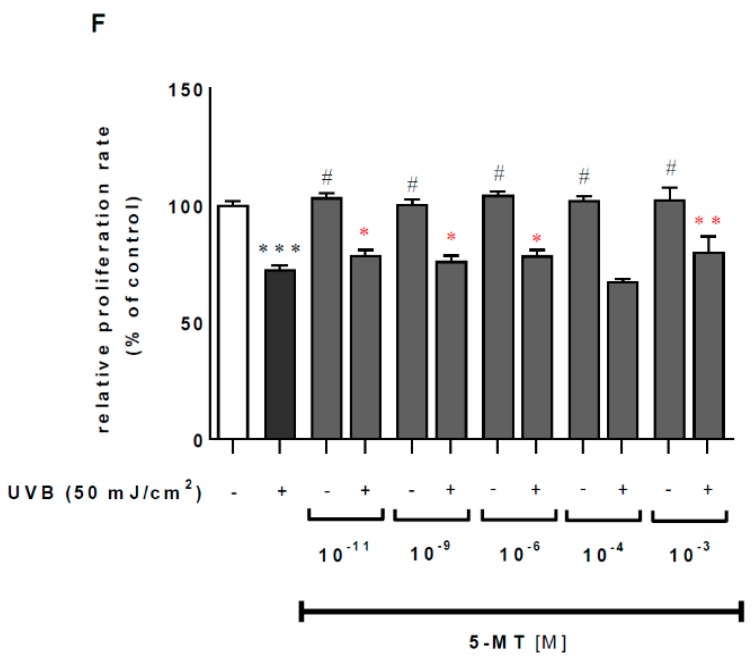
Ultraviolet radiation (UVR)-mediated reduction of viability is attenuated by melatonin, 6-hydroxymelatonin (6(OH)Mel), and 5-methoxytryptamine (5-MT) in MNT-1 melanoma cells. Ultraviolet B (UVB)-irradiated (50 mJ/cm^2^) and non-irradiated cells (presented as inserts) were treated with graded concentrations of melatonin and its selected metabolites for 1 h prior to UVR. Viability was determined 48 h post-UVR by MTT assay (**A**,**C**,**E**) and crystal violet assessment (**B**,**D**,**F**), as described in the Section 4. Values were expressed as a percentage of the UVR-irradiated (50 mJ/cm^2^) or sham-irradiated sample (insert). Statistically significant differences versus control were indicated as * *p* < 0.05, ** *p* < 0.01, *** *p* < 0.001; with (^#^ *p* < 0.001) indicating a significant difference between sham-irradiated cell and UVR-exposed samples at particular concentrations. Red labeling indicates the effect of presence of tested compounds compared to UV-treated cells.

UVB induces serious intracellular disturbances such as DNA damage and the production of ROS, leading to cell mortality or carcinogenesis. Firstly, we assessed the effect of UVB on the viability of MNT-1 cells, and noticed a decrease in cell viability of approximately 52% (*p* < 0.001) compared to the sham-irradiated controls, while melatonin as well as its metabolites themselves did not affect the survival rate of MNT-1 cells, even at the highest tested concentration (10^−3^ M) (Figure 1, insert). Subsequently, the dose-dependent (10^−11^–10^−3^ M) analysis was performed for melatonin (Mel) (Figure 1A), 6-hydroxymelatonin (6(OH)Mel) (Figure 1CE) under ultraviolet B (UVB) exposure. Pre-incubation with all three agents protected UVB-irradiated cells at the physiologic range of day and night plasma levels, i.e. a concentration of 10^−11^ M by 8% (*p* < 0.05; Mel), 24% (*p* < 0.001; 6(OH)Mel), and 19% (*p* < 0.001; 5-MT) or by 6% (*p* < 0.05; Mel), 13% (*p* < 0.01; 6(OH)Mel), and 13% (*p* < 0.05; 5-MT) for 10^−9^ M. Middle-range doses (10^−8^–10^−6^ M) still revealed the protective action of the tested compounds; however, significant differences were moderate or none (10^−6^ M) in all of the cases. Finally, the pharmacological doses (10^−4^ or 10^−3^ M) ameliorated a decrease in cell viability compared to the UVR-treated cells by 13% (*p* < 0.01; Mel), 12% (*p* < 0.01; 6(OH)Mel), and 9% (*p* < 0.05; 5-MT) for 10^−3^ M. These data were also supported by crystal violet assessment, where UVB caused a dramatic drop of MNT-1 proliferation ratio by 34% (*p* < 0.001) compared to the control cells, and pre-incubation with Mel (Figure 1B), 6(OH)Mel (Figure 1D), or 5-MT (Figure 1F) significantly counteracted this effect. 

### 2.2. Melatonin and Its Metabolites Protect MNT-1 Cells from UVB-Induced Oxidative Stress and Disturbances in Calcium Homeostasis

**Figure 2:**
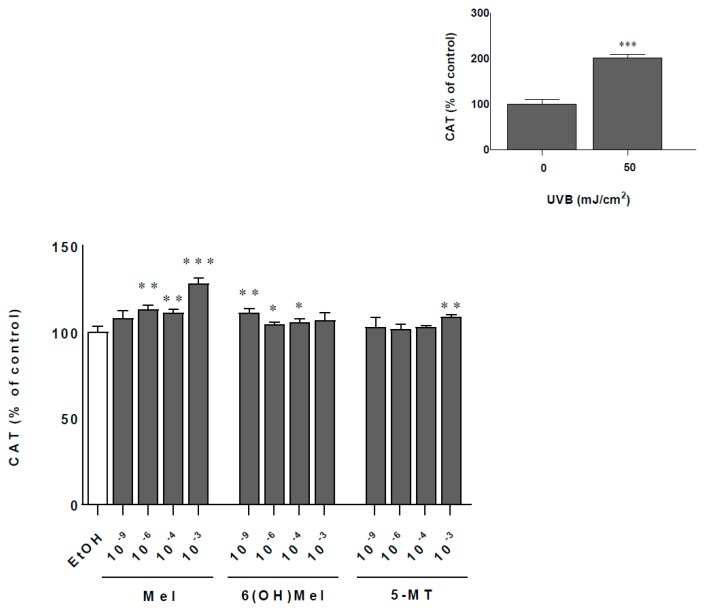
Melatonin, 6(OH)Mel, and 5-MT affect catalase activity (CAT) under UVR-induced stress condition in MNT-1 cells. Ultraviolet B (UVB)-irradiated (50 mJ/cm^2^) cells were pre-treated with graded concentrations of melatonin and its selected metabolites for 1 h prior to UVR. CAT activity was determined 48 h post-UVR by the colorimetric assay as described in the Section 4. Values were expressed as percentage of the UVR-irradiated (50 mJ/cm^2^) or sham-irradiated sample (insert). Statistically significant differences were indicated as * *p* < 0.05, ** *p* < 0.01, *** *p* < 0.001.

**Figure 3:**
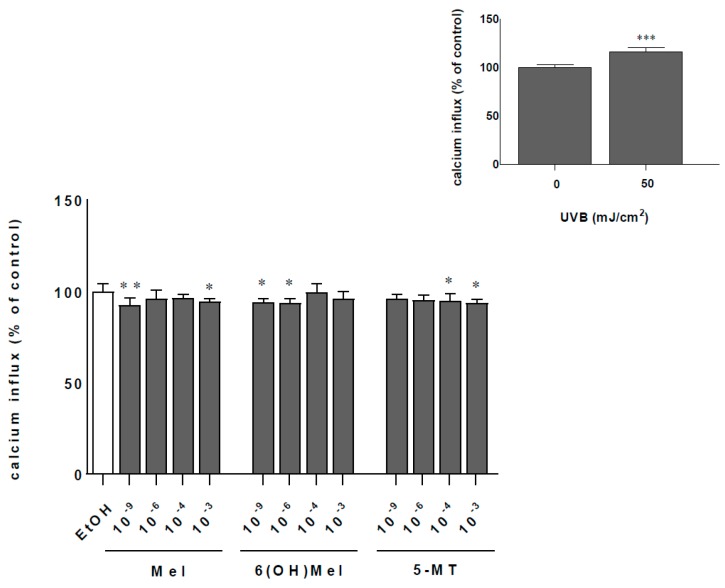
UVR-mediated disturbances in Ca^++^ homeostasis are attenuated by melatonin, 6(OH)Mel, or 5-MT in MNT-1 cells. UVB-irradiated (50 mJ/cm^2^) were pre-treated with graded concentrations of melatonin and its selected metabolites for 1 h prior to UVR. Calcium influx was determined 48 h post-UVR by the colorimetric assay, as it was described in the Section 4. Values were normalized and expressed as a percentage of the UVR-irradiated (50 mJ/cm^2^) or sham-irradiated sample (insert). Statistically significant differences were indicated as * *p* < 0.05, ** *p* < 0.01, *** *p* < 0.001.

Cells exposed to 50 mJ/cm^2^ UVB showed a twofold increase (*p* < 0.001) of catalase activity (CAT) activity compared to sham-irradiated samples (Figure 2; insert). Additionally, comparative analysis of melatonin actions revealed the strongest enhancing effect at a concentration of 10^−3^ M Mel by 28% (*p* < 0.001) compared to the control. At lower concentrations, this impact was less pronounced, e.g., 11% (10^−4^ M), 13% (10^−6^ M) (*p* < 0.01), and 9% (10^−9^ M; not significant). The presence of metabolites of melatonin enhanced significantly CAT activity by 11% (10^−9^ M; *p* < 0.01) or by 9% (10^−3^ M; *p* < 0.01) for 6(OH)Mel and 5-MT, respectively (Figure 2). UVB irradiation induced a massive calcium influx with 16% (*p* < 0.001) more calcium inside the cell compared to the non-irradiated cells (Figure 3; insert). Pre-incubation for 1 h with melatonin or its metabolites counteracted these effects modestly by 8% (10^−9^ M Mel; *p* < 0.01); 6% (10^−9^ M 6(OH)Mel; *p* < 0.05), and 4% (10^−9^ M 5-MT; not significant). The highest concentration (10^−3^ M) of the compound showed a similar pattern of regulation arresting calcium influx by 6%, 5%, and by 8%, respectively, for melatonin (*p* < 0.05), 6(OH)Mel, and 5-MT (*p* < 0.05) (Figure 3). 

### 2.3. Melatonin and Its Metabolites Maintain Mitochondrial Function 

**Figure 4:**
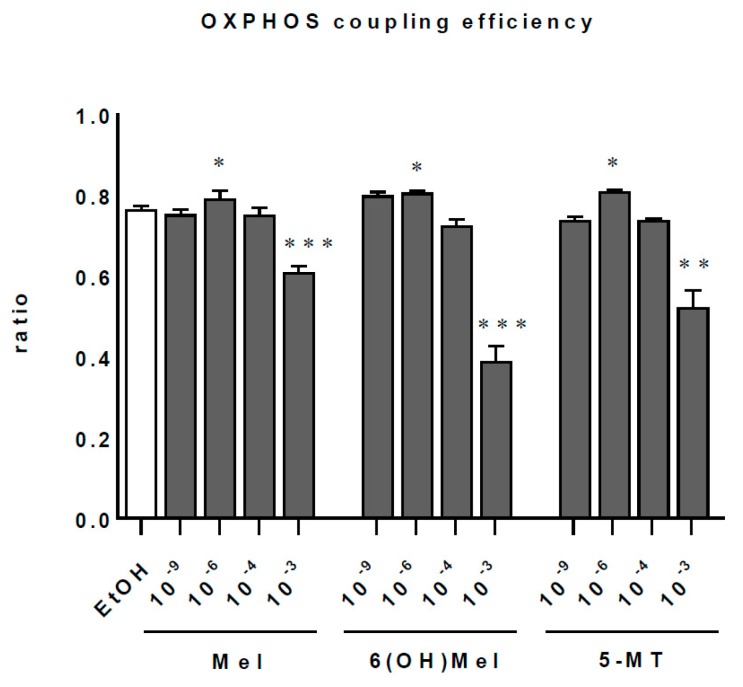
Effect of melatonin and its selected metabolites on bioenergetics. Parallel analysis of mitochondrial coupling efficiency (OXPHOS coupling efficiency) measured in liver mitochondria incubated without (control) or in presence of Mel, 6(OH)Mel, 5-MT. Results are presented as the means + SEM. Statistically significant differences compared to mitochondria without tested compounds (control) are as follows: * *p* < 0.05, ** *p* < 0.01, *** *p* < 0.001.

**Figure 5:**
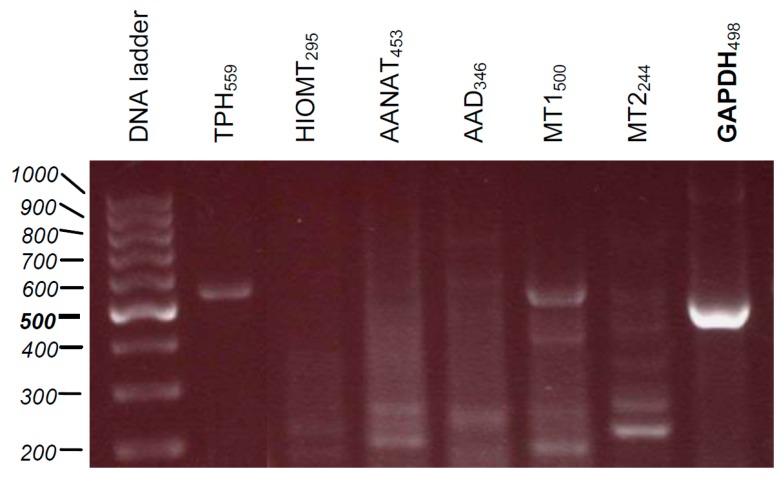
Evaluation of expression of enzymes regulating cutaneous melatonin biosynthetic pathway (tryptophan-5-hydroxylase (TPH1) and hydroxyindole-*O*-methyl transferase (HIOMT)) as well as melatonin membrane receptors (MT1 and MT2) in parallel to housekeeping gene glyceraldehyde phosphate dehydrogenase (GAPDH) in intact MNT-1 cells using specific primers (Table 2).

Since melatonin penetrates very easily through cellular membranes reaching numerous intracellular organelles, we assessed the influence of melatonin and its selected metabolites on mitochondrial function (Figure 4; Table 1). These analysis identified Mel, 6(OH)Mel, and 5-MT as the compounds enhancing markedly (*p* < 0.01) oxidative phosphorylation at the dose of 10^−6^ M with subtle (not significant) differences at 10^−9^ or 10^−4^ M. Surprisingly, the highest dose of 10^−3^ M for all tested substances decreased prominently the OXPHOS coupling efficiency (*p* < 0.001 or *p* < 0.01). Evaluation of the mitochondrial metabolic states (Table 1) showed that in “native” conditions (without stress), lower concentrations (10^−9^ or 10^−6^ M) of Mel, 6(OH)Mel, and 5-MT are more optimal than the pharmacological ones for maintenance of the mitochondrial homeostasis. MNT-1 cells express enzymes that play key roles in the synthesis of melatonin, including tryptophan-5-hydroxylase (TPH), and hydroxyindole-*O*-methyl transferase (HIOMT), and show the presence of MT1 and MT2 (Figure 5). This is consistent with previous reports on the capability of human melanoma cells to produce and metabolize melatonin [46] and the expression of TPH1 in human melanoma skin biopsies [47] and rodent melanomas [48,49]. 

## 3. Discussion

Cutaneous cells are under the constant influence of multiple environmental stressors [50], including UVR, which is the main source of generation of reactive oxygen/nitrogen species (ROS/RNS), leading to deleterious oxidative skin damage [40,42,51] that includes cell senescence, photoaging, carcinogenesis, or inflammation. Thus, searching for the sun damage-preventing natural compounds that can counteract UVB-induced increase in levels of ROS/RNS is strongly desired. One of such compounds is melatonin, which possesses enhancing capacities regarding skin protection against UVR [29,32,52]. It is commonly known as a free radical scavenger [53,54]; it detoxifies NO [55], H_2_O_2_, O_2_●^−^ [56,57], and OH● [58]. To date, studies have shown the potential beneficial effects of melatonin in the treatment of many chronic diseases, while it exhibits low or absent toxicity [59]. Additionally, it exhibits Nrf2-mediated protective effects in animal models [23] or cutaneous cells [27,33]. Apart from its significant anti-oxidative properties, melatonin also preserves mitochondrial function in different in vitro and in vivo models [23,60,61,62]. Previous reports [63,64] claim that melatonin should be considered as a critical modulator of mitochondrial integrity and physiology in skin cells [26], and this function can be also assigned to its metabolites [65]. This assumption has been substantiated in several mitochondrial-related disorders such as murine models of Parkinson’s disease [66,67,68], and the direct action of melatonin and metabolites on mitochondrial homeostasis [69,70]. This experimental evidence identifies mitochondria as a likely site of melatonin synthesis [71], placing this compound as a strategic molecule during the evolution of aerobic eukaryotes [72]. Our data confirmed that melatonin and its selected metabolites can protect cells from UVB-mediated oxidative stress by enhancing the activity of catalase, decreasing Ca^++^ influx, and maintaining mitochondrial function. It should be still noted that melatonin, 6(OH)Mel, or 5-MT, particularly at pharmacological doses (10^−3^ M), seem to affect mitochondrial function in the absence of any exogenous stressors (Figure 4). Interestingly, these concentrations were found to be the most protective under UVB treatment. Thus, we assume that in normal conditions at higher concentrations (10^−3^/10^−4^ M), the mitochondrial fission could occur, but this hypothesis remains to be clarified. On the other hand, we propose that melatonin may act as a sort of “standby molecule” or “cellular guardian” whose anti-oxidative capacities are activated in case of cell stress. Indeed, it was reported in rat heart mitochondria that melatonin inhibits stress-induced mitochondrial permeability transition pore (mPTP) opening and release of cytochrome c [73,74]. It is also known that treatment with melatonin at a pharmacological dose (10^−3^ M) affects mitochondrial function [75,76], which is consistent with our results performed on isolated mitochondria. Moreover, Odinokova et al. [77] showed that melatonin drastically decreases the expression of complex I subunit, which is accompanied by the suppression of ROS production and the inhibition of mPTP opening. As we noticed here, the mechanism of action of melatonin is complexed, and G protein-coupled melatonin type 1 and type 2 receptors (MT1 and MT2) as well as key enzymes (TPH1 and HIOMT) for its synthesis are expressed in MNT-1 cells. According to the earlier detections, melatonin receptors have been reported in melanocytes (normal and malignant), keratinocytes (normal), or dermal fibroblasts [30,78,79,80,81]. The expression of both MT1 and MT2 receptors in MNT-1 melanoma cells suggests that they might be involved in a regulation of calcium homeostasis in this cell line under stress conditions. However, it is well described that calmodulin is another melatonin-binding protein, which may be physiologically relevant to affinity after Ca^++^ binding and interaction with calmodulin-controlled enzymes, including calmodulin kinase II (CaMKII) and calcineurin (Cn), which regulate intracellular calcium homeostasis [11,82]. These observations may partially explain the role of melatonin and its metabolites in the endoplasmic reticulum stress response, as well as the regulation of apoptosis, autophagy, and mitochondrial function [83]. In addition, Martin et al. [62] observed that melatonin arrested ruthenium red (RR)-mediated oxidative stress and mitochondrial uncoupling where RR is a molecule that inhibits the mitochondrial Ca^++^ uniporter. Also, our previous experiments [28,84] showed that melatonin in the skin is rapidly metabolized and/or degraded through both indolic and kynuric pathways. The indolic pathway produces 6(OH)Mel as the main product and 5-MT as a minor one, and these two metabolites of melatonin are produced in skin cells [28]; similar effects are also observed in the kidney. Thus, the predominant pathway of melatonin metabolism in all of the main skin cell populations (human normal epidermal keratinocytes and melanocytes, human normal dermal fibroblasts) and malignant melanoma cells is similar to that described at the systemic level, in which 6(OH)Mel is the main degradation product [65].

**Figure 6:**
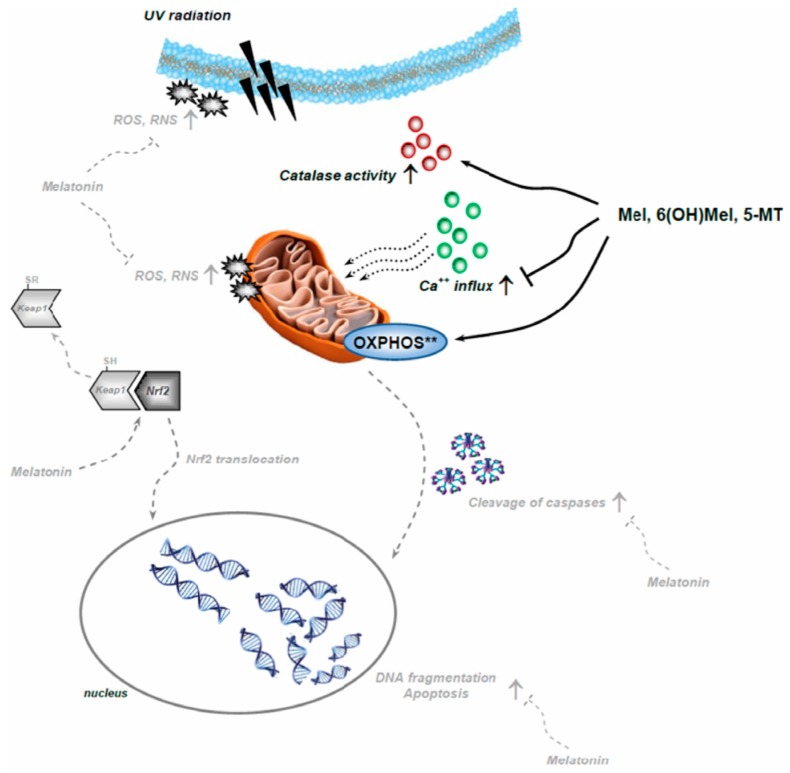
Schematic diagram of the complex mechanisms of inductive (arrows) and inhibiting (T line) action of melatonin counteracting UVR-induced intracellular alterations based on earlier reports [25,27,28,32,33,44] (labeled in grey and dashed lines) with modification based on the presented data (labeled in black). ** - experiments conducted in isolated mitochondria (without UV exposure).UVR, reported as one of the main deleterious environmental skin stressors, is prominently counteracted and/or modulated by melatonin in the context of a complex intracutaneous melatoninergic anti-oxidative system with UVR-enhanced or UVR-independent melatonin metabolites [29,52]. Therefore, endogenous intracutaneous melatonin production, together with topically applied exogenous melatonin or metabolites would be expected to represent anti-oxidative defense systems against the UVB-induced skin damages [13,52]. In summary, a key question is whether melatonin maintaining mitochondrial homeostasis can be exploited therapeutically as a protective agent, as a general “skin survival factor” or as a “defender” of the genome and cellular integrity with clinical applications in UVR-induced pathology, including carcinogenesis and skin aging.

As mentioned above, mitochondria are one of the main target organelles for melatonin and play the key role in disease initiation and/or progression. Although restoring mitochondrial function and redox balance/signaling is not a straightforward process, mitochondria can convert external stimuli into alterations in antioxidant defense mechanisms and energetic demands, making cells resistant to stress. This phenomenon is known as mitohormesis, and is detrimental for restoring mitochondrial function and control/regulation in non-physiological mtROS production. Since melatonin and its metabolites over the years have been described as effective anti-inflammatory, anti-oxidative and anti-apoptotic agents, also under UVR conditions (Figure 6), the development of novel mitochondria-targeted multi-functional antioxidants based on melatonin are of great interest in skin biology and physiology [11,26]. These compounds maintaining mitochondrial homeostasis by direct scavenging mtROS can increase cell resistance to stress and viability; therefore, it should be taken under consideration as possible future drugs to improve mitochondrial health in primary and/or secondary mitochondrial-related diseases.

## 4. Materials and Methods 

### 4.1. Reagents

Dulbecco’s modified Eagle’s medium (DMEM) with high glucose (4500 mg/L), 1% penicillin-streptomycin solution (10,000 units of penicillin and 10 mg of streptomycin in 1 mL 0.9% NaCl), EGTA, ethanol, HEPES (1 M), isopropanol, KH_2_PO_4_, mannitol, MgCl_2_·6H_2_O, MTT, non-essential amino acids (NEAA) (100×), l-glutamic acid, l-malic acid, potassium lactobionate, sodium pyruvate, sodium pyruvate solution (100 mM), succinic acid, and taurine were purchased from Sigma (St. Louis, MO, USA). Fetal bovine serum, 0.05% trypsin/0.53 mM EDTA solution, 1× PBS (pH 7.4), l-glutamine (200 mM) were supplied by Thermo Fisher Scientific (Waltham, MA, USA). 

### 4.2. Cell Culture

MNT-1 human melanotic cells were acquired as a gift from Dr. Cédric Delevoye (Institute Curie, Paris, France). Cells were maintained in T-75 or T-175 flasks in DMEM medium supplemented with 20% (*v*/*v*) heat-inactivated fetal bovine serum, 2 mM of l-glutamine, 10 mM of HEPES, 1 mM of sodium pyruvate, 1% (*v*/*v*) NEAA, and 1% (*v*/*v*) streptomycin-penicillin solution at 37 °C in a humidified atmosphere of 5% CO_2_ in air. Cells in the logarithmic growth phase were used in all of the experiments, while 80–90% monolayers of confluent MNT-1 cells were harvested with a mixture of 0.05% trypsin–EDTA solution.

### 4.3. Pre-incubation with Melatonin, Its Metabolites, and UV Irradiation

Prior to the start of the experimental treatments, cells were cultured in normal medium for 24 h in order to attach to the bottom of the culture dish. Thereafter, culture medium was replaced with medium containing Mel, 6(OH)Mel, 5-MT, or solvent (control) (Sigma). Compounds were dissolved in absolute ethanol and further diluted with phosphate-buffered saline (1× PBS, pH 7.4) to yield 10^−2^ M stock solution, then diluted 1:10 with culture medium to yield a final wide concentration range of powers of 10 from 10^−3^ to 10^−11^ M for cell culture pre-incubation. Melatonin-free, 6(OH)Mel-free, or 5-MT-free medium served as controls. After 1 h pre-incubation with test compounds, cells were washed twice with 1× PBS to remove remnants of medium, and fresh 1× PBS was added prior to UVR exposure. Irradiation was performed using an irradiation bank consisting of six UV bulbs (TL12, Philips, The Netherlands) that emit most of their energy within the UVB range (280–320 nm) with an emission peak of 313 nm. UVB doses of 0 and 50 mJ/cm^2^ were used, and UVR-treated cells were examined 48 h post-UVR. Sham-irradiated samples (0 mJ/cm^2^) were prepared using a culture dish covered with aluminum foil and were considered as the control value. After UVR exposure, cells were cultured and then subjected to the particular measurements.

### 4.4. MTT Viability Assay 

Tested cells were seeded on 96-well plates at the density of 0.15 × 10^5^ cells/well in the culture medium, and thereafter grown to subconfluence (as judged from light microscopy). Following the pre-incubation with tested compounds, cells were UV-irradiated in presence of 100 μL of pre-warmed 1× PBS, and then cultured until the desired time end-point. Cell viability was evaluated by MTT assay based on the protocol described earlier by Carmichael et al. [85]. MTT (5 mg/mL) was dissolved in 1× PBS, sterilized by filtration through a 0.22-μM Millipore^®^ filter and stored at 4 °C. After treatment as described above, cells were washed twice with 1×PBS; then, 100 μL of MTT solution in culture medium (the final dilution, 1:10) was added to each well. The cells were subsequently incubated for 3 h at 37 °C to allow MTT metabolism. The formazan produced was dissolved in 100 μL of isopropanol/0.04 N HCl, and absorbance was measured at 595 nm using a BioTek ELx808™ microplate reader (BioTek Instruments, Inc., Winooski, VT, USA). 

### 4.5. Crystal Violet Assessment

The proliferation of MNT-1 cells was assessed using ready-to-use crystal violet (CV) solution (Cell Biolabs, Inc., San Diego, CA, USA). Cells were seeded on six-well plates (0.4–0.5 × 10^6^ cells/cm^2^) and experiments were performed when cultures reached 70–80% confluence. At the desired times, cells were washed twice with 1× PBS, stained with 0.1% CV solution in 10% ethanol for 1 min, and remnants were washed again with 1× PBS. Plates were photographed and the quantification of cells was performed using ImageJ 1.51f software (National Institute of Health, Bethesda, MD, USA). 

### 4.6. Catalase Activity Assay

Evaluation of catalase (CAT) activity was conducted by colorimetric assays (BioVision Research Products, Inc., Mountain View, CA, USA). Briefly, cells were seeded on six-well plates at the density and cultured until reached confluency as described in point 4.5. At the desired times, cells were lysed with catalase assay buffer and then centrifuged for 15 min at 10,000× *g* at 4 °C. Resultant supernatant was proceeded for catalase reaction with one mM of H_2_O_2_ for 30 min at 25 °C, and arrested subsequently with a stop solution. Catalase activity was determined by incubation at room temperature (RT) for 15 min with a develop mix containing catalase assay buffer, OxiRed™ Probe, and HRP solution. The absorbance of tested samples was measured at 595 nm using a BioTek ELx808™ microplate reader (BioTek Instruments, Inc., Winooski, VT, USA).

### 4.7. Calcium Assay

Intracellular calcium homeostasis was assessed by colorimetric assay supplied by BioVision Research Products, Inc. After reaching desired confluency, cells were lysed and centrifuged for five min at 16,000× *g* at 4 °C. The resultant supernatant was mixed with the chromogenic reagent, calcium assay buffer, and then incubated at RT for 15 min protecting from light. The absorbance of tested samples was measured at 595 nm using a BioTek ELx808™ microplate reader (BioTek Instruments, Inc.)

### 4.8. Animals and Isolation of Liver Mitochondria 

All of the experimental procedures were performed in accordance with the principles of the European animal research laws (European Communities Council Directive 2010/63/EU). Wild-type female BALB/cJ mice (*n* = 8; weight: 20–25 g) were kept in an environmentally controlled room (23 ± 1 °C with a 12-h light-dark cycle) receiving standard mice chow and water ad libitum. The mice were euthanized by cervical dislocation. The liver mitochondria were isolated, as previously described by Broekemeier et al. [86] with slight modification [87]. The liver was rapidly removed, weighed, and placed in ice-cold mitochondrial buffer A (230 mM of mannitol, 70 mM of sucrose, three mM of HEPES, 0.1 mM of EGTA; pH 7.4), and subsequently rinsed three times with buffer A. The liver was minced with scissors and homogenized using a Teflon pestle homogenizer in buffer A (20 mL/1 g of liver). The homogenate was centrifuged at 800× *g* for 10 min; then, the supernatant was decanted and centrifuged at 4000× *g* for 10 min. The pellet was resuspended in 30 mL of suspension buffer B (230 mM of mannitol, 70 mM of sucrose, 3 mM of HEPES), and centrifuged again at 7000× *g* for 10 min. This step was repeated twice. The final mitochondrial pellet was resuspended in 0.5 mL of buffer B. All of the steps were performed at 4 °C. Total protein concentration (20 to 30 mg of mitochondrial protein per mL) was determined with the Bradford reagent (Bio-Rad Laboratories, Inc., Hercules, CA, USA) [88].

### 4.9. High-Resolution Respirometry

Respiration was measured at 37 °C under constant stirring (750 rpm), which ensured a homogenous oxygen distribution in the medium in a high-resolution respirometer using an Oxygraph-2k (O2k, Oroboros Instruments, Innsbruck, Austria), a modular system for high-resolution respirometry (HRR) according to Volani et al. [89]. Oxygen concentration (μM) and oxygen flux per mass (pmol O_2_● s^−1^·mg^−1^) were recorded in real-time, while obtained data were evaluated using DatLab software (Oroboros Instruments, Innsbruck, Austria). Briefly, mitochondrial respiration was measured in MiR05 (mitochondrial respiration medium containing 0.5 mM of EGTA, 3 mM of MgCl_2_·6H_2_O, 60 mM of potassium lactobionate, 20 mM of taurine, 10 mM of KH_2_PO_4_, 20 mM of HEPES, 110 mM of sucrose, and 1 g/L of BSA fatty acid-free (pH 7.1) (MiR05; Oroboros Instruments, Innsbruck, Austria). Freshly isolated liver mitochondria (0.1 mg of mitochondrial protein per each 2-mL chamber) used for respiration measurement were first incubated for 5 min in the medium without (control) or in presence of Mel, 6(OH)Mel, or 5-MT. 

For the assessment of mitochondrial respiration, the following protocol was used:

(1) Non-phosphorylating LEAK respiration was assessed by injecting 10 mM of sodium pyruvate, 10 mM of l-glutamic acid (neutralized with KOH) and 2 mM of l-malic acid (neutralized with KOH) as NADH (N)-linked substrates: state N*L*;
(2) OXPHOS capacity was induced by adding 1.25 mM of ADP at saturating concentration: state N*P*;
(3) 10 μM of cytochrome c was added to test the integrity of the outer mitochondrial membrane: state N*C*;
(4) NADH and succinate (NS)-linked OXPHOS capacity was measured by adding 10 mM of succinic acid (neutralized with KOH): state NS*P*.

### 4.10. Mitochondrial Quality and Control

The OXPHOS coupling efficiency was calculated as measures of mitochondrial quality and control. OXPHOS coupling efficiency, calculated with the formula (1)—(state N*L*)/(state N*P*), reflects the coupling of respiration supported by electron transferring flavoprotein (ETF) with pyruvate, glutamate, and malate as substrates before (state N*L*) and after the addition of ADP (state N*P*). The OXPHOS coupling efficiency (*OCE*) can be compared with the respiratory control ratio (*RCR*). The formula of *OCE* could be rewritten as:

(1) $$OCE=\left(1-\frac{1}{RCR}\right)$$

The *OCE* is noted between 0 and 1, while the *RCR* could be from 0 to infinite. A lower value of *OCE* means lesser coupling of the oxidation and phosphorylation after the addition of ADP. As the *OCE* decreases, it is therefore less coupled [90]. 

### 4.11. RNA Isolation, cDNA Synthesis, and PCR

RNA from MNT-1 cell pellet (5 × 10^6^ cells) was extracted accordingly to the manufacturer’s instructions using the innuPREP RNA Mini Kit (Analytik Jena, Berlin, Germany). The amount of RNA was determined using BioPhotometer (Eppendorf, Hamburg, Germany). cDNA synthesis was conducted using RevertAid™ First Strand cDNA Synthesis Kit (Thermo Fisher Scientific, Waltham, MA, USA) in the presence of oligo(dT) primers as follows: 65 °C for 5 min, 42 °C for 60 min, 70 °C for 5 min in Thermomixer (Eppendorf), and the resultant cDNA was stored at −20 °C prior to PCR reaction. Reactions were carried out by GoTaq^®^ PCR Master Mix (Promega GmbH, Mannheim, Germany) in the presence of sequences of primers (Table 2). Amplification was performed using 10 min of initial denaturation at 95 °C followed by three-step 39-cycling of 60 s at 95 °C (denaturation), 60 s at 60 °C (annealing), and 60 s at 72 °C (extension). PCR products were separated on 1.8% agarose gel containing RedSafe™ Nucleic Acid Staining solution (iNtRON Biotechnology, Sangdaewon-Dong, Korea), and afterwards visualized using the Fusion-FX7 UV transilluminator (Vilber GmbH, Eberhardzell, Germany). Samples were standardized by amplification of the housekeeping gene glyceraldehyde phosphate dehydrogenase (GAPDH) [12]. DNA-standard O’GeneRuler™ 100 bp DNA Ladder Mix from Fermentas International (Burlington, ON, Canada) was used. 

### 4.12. Statistical Analysis

Data were expressed as pooled means (at least *n* = 3) ±standard error of the mean (SEM). The data were analyzed using *t*-Test (for two groups) or with one-way ANOVA (for multiple groups) with GraphPad Prism 7.05 software (La Jolla, CA, USA). Obtained data for MTT viability assay, crystal violet assessment, CAT activity, or Ca^++^ homeostasis were normalized and expressed as a percentage of the control value i.e., sham-irradiated sample (0 mJ/cm^2^, inserts) or UVB-exposed samples. The results of mitochondrial metabolism were presented in arbitrary units. A *p*-value of less than 0.05 was considered statistically significant. 

## Figures and Tables

**Table 1:** Effect of melatonin and its metabolites on the bioenergetics of mitochondria. Non-phosphorylating LEAK respiration (state NL), OXPHOS capacity (state NP), integrity of the outer mitochondrial membrane: (state NC), and NADH and succinate (NS)-linked OXPHOS capacity (state NSP) were measured in liver mitochondria incubated without or in presence of melatonin (Mel), 6(OH)Mel, or 5-MT. Results are presented as the mean ± SEM. Statistically significant differences compared to mitochondria without tested compounds (control) were presented as follows: * *p* < 0.05, ** *p* < 0.01, *** *p* < 0.001.

	EtOH (control)	10^−9^ M	10^−6^ M	10^−4^ M	10^−3^ M
**State N*L***					
**Melatonin**	14.95 ± 0.50	12.87 ± 0.84 *	12.76±1.25 *	14.60±0.94	18.13±1.03
**6-OH-melatonin**		15.31 ± 0.70	14.40 ± 0.54	16.45 ± 0.65	18.97 ± 0.97
**5-MT**		12.80 ± 0.39 **	11.97 ± 0.32 **	16.91 ± 0.48	24.25 ± 0.88 **
**State N*P***					
**Melatonin**	79.04 ± 2.34	64.90 ± 2.34 *	74.19 ± 2.85	73.84 ± 3.50	64.56 ± 3.16 *
**6-OH-melatonin**		91.75 ± 1.02 *	88.82 ± 2.75 *	76.72 ± 2.55	50.27 ± 1.79 **
**5-MT**		62.16 ± 2.52 **	74.67 ± 0.81	81.61 ± 2.14	75.62 ± 1.72
**State N*C*** (% of corresponding state N*P*)					
**Melatonin**	121.10 ± 2.01	123.80 ± 1.15	116.30 ± 0.73	119.10 ± 1.83	112.80 ± 1.27
**6-OH-melatonin**		118.30 ± 0.74	125.20 ± 2.44	150.60 ± 1.61 ***	203.60 ± 15.11 **
**5-MT**		124.80 ± 1.68	115.20 ± 4.27	119.60 ± 0.93	116.00 ± 0.93
**State NS*P***					
**Melatonin**	300.00 ± 9.64	248.90 ± 8.65 *	262.00 ± 8.30	267.30 ± 12.61	266.10 ± 9.90
**6-OH-melatonin**		327.10 ± 3.51 *	328.00 ± 3.95 **	368.90 ± 5.22 ***	311.60 ± 9.66
**5-MT**		260.00 ± 9.53	280.60 ± 3.97	313.30 ± 1.30	295.90 ± 3.41

**Table 2:** The primers used for PCR, oriented 5’→3’.

Gene	Primer Sequences	Product Size (bp)
***HIOMT***	F: TTCCAGGAAGGGGATTTCT R: GAAGCCAGCAGAAGAGAGGA	295
***MT1***	F: GCGTCCTCATCTTCACCATC R: GACGAGGAAGTGGAAAACCA	500
***MT2***	F: TATCACTGCCATCGCCATTA R: GAGGAGGAAGTGGATGACCA	244
***TPH***	F: GACAACGTCCCCCATACTCT R: CATAGCCAAGTCCGCAAAAT	559
***GAPDH***	F: AAGGTCATCCCTGAGCTGAA R: CCCCTCTTCAAGGGGTCTAC	498

## Abbreviations

5-MT	5-Methoxytryptamine
6(OH)Mel	6-Hydroxymelatonin
CAT	Catalase
DMEM	Dulbecco’s modified Eagle’s medium
HIOMT	Hydroxyindole-*O*-methyl transferase
HRR	High-resolution respirometry
Mel	Melatonin
mPTP	Mitochondrial permeability transition pore
*OCE*	OXPHOS coupling efficiency
*RCR*	Respiratory control ratio
RNS	Reactive nitrogen species
ROS	Reactive oxygen species
TEM	Transmission electron microscopy
TPH	Tryptophan-5-hydroxylase
UVR	Ultraviolet radiation
